# The Generation of Turnip Crinkle Virus-Like Particles in Plants by the Transient Expression of Wild-Type and Modified Forms of Its Coat Protein

**DOI:** 10.3389/fpls.2015.01138

**Published:** 2015-12-24

**Authors:** Keith Saunders, George P. Lomonossoff

**Affiliations:** Department of Biological Chemistry, John Innes Centre, Norwich Research ParkNorwich, UK

**Keywords:** turnip crinkle virus, virus-like particles, infiltration, agrobacterium, plant expression, fusion protein, encapsidation

## Abstract

Turnip crinkle virus (TCV), a member of the genus *carmovirus* of the *Tombusviridae* family, has a genome consisting of a single positive-sense RNA molecule that is encapsidated in an icosahedral particle composed of 180 copies of a single type of coat protein. We have employed the CPMV-*HT* transient expression system to investigate the formation of TCV-like particles following the expression of the wild-type coat protein or modified forms of it that contain either deletions and/or additions. Transient expression of the coat protein in plants results in the formation of capsid structures that morphologically resemble TCV virions (*T* = 3 structure) but encapsidate heterogeneous cellular RNAs, rather than the specific TCV coat protein messenger RNA. Expression of an amino-terminal deleted form of the coat protein resulted in the formation of smaller *T* = 1 structures that are free of RNA. The possibility of utilizing TCV as a carrier for the presentation of foreign proteins on the particle surface was also explored by fusing the sequence of GFP to the C-terminus of the coat protein. The expression of coat protein-GFP hybrids permitted the formation of VLPs but the yield of particles is diminished compared to the yield obtained with unmodified coat protein. Our results confirm the importance of the N-terminus of the coat protein for the encapsidation of RNA and show that the coat protein's exterior P domain plays a key role in particle formation.

## Introduction

Virus-like particles (VLPs) are increasingly being utilized as vaccines and are now finding use in emerging roles in the rapidly and ever expanding bionanotechnology field. Essentially, VLPs are identical in structure and morphology to virus particles but, unlike virus particles, they do not contain infectious nucleic acid. Consequently, their use as potential vaccines has been and continues to be extensively studied (Bachmann and Jennings, 2010). Because of their size and functionality, roles for modified VLPs in cancer therapies, for example as potential drug delivery vehicles and cancer-targeted imaging agents, are under active study (Cho et al., 2014). In recent times VLPs synthesized via plant-based expression systems, as opposed to eukaryotic cell culture and bacterial expression systems, have demonstrated considerable potential to satisfy the ever increasing demand for VLPs for a number of purposes. Consequently plant RNA virus particles, and modified forms thereof, have often been the starting point for a variety of studies, their abundant accumulation and stability providing a vital prerequisite for subsequent studies and applications (Lomonossoff and Evans, 2011).

Plant virus particles produced via infection, and therefore containing the viral genome, have been investigated as nanoparticles for over 20 years. For example, epitope display on the surface of a spherical plant virus, *cowpea mosaic virus* (CPMV), was first demonstrated in the early 1990s (Usha et al., 1993; Porta et al., 1994) and a number of other plant viruses, of various morphologies were subsequently used for this purpose (for reviews, see Porta and Lomonossoff, 1998; Lomonossoff, 2010; Lomonossoff and Evans, 2011). Although successful, the use of infectious particles has a number of limitations: there are problems of biocontainment and the presence of the viral genome within the particles can interfere with their loading with heterologous material other than small molecules unless the particles are first disassembled and the genomic material removed. Furthermore, there are limits to the changes which can be made to the surface of the particles without adversely affecting virus infectivity. For these reasons, attention shifted to the expression of the coat proteins of a number of plant viruses in a variety of heterologous systems, such as bacteria, yeast and insect cells, to obtain VLPs through the ability of the subunits to self-assemble (Young et al., 2008). It was not until the development of an efficient transient expression system that it was possible to investigate the use of plants for the efficient expression of large quantities of plant virus-derived VLPs. The first example of the successful production genome-free plant virus-based VLPs in plants concerned CPMV; in this case these were readily synthesized by the expression of the CPMV capsid protein precursor protein (VP60) in combination with the CPMV 24K protease (Saunders et al., 2009). Without the constraints of viral infection to generate particles, there are an increasing number of modifications to the CPMV capsid surface protein that can now be made. In contrast to RNA-containing particles, it is possible to load CPMV VLPs with a variety of foreign materials (Aljabali et al., 2010) thereby exploiting their potential for many future medical and bionanotechnological applications.

**Figure 1:**
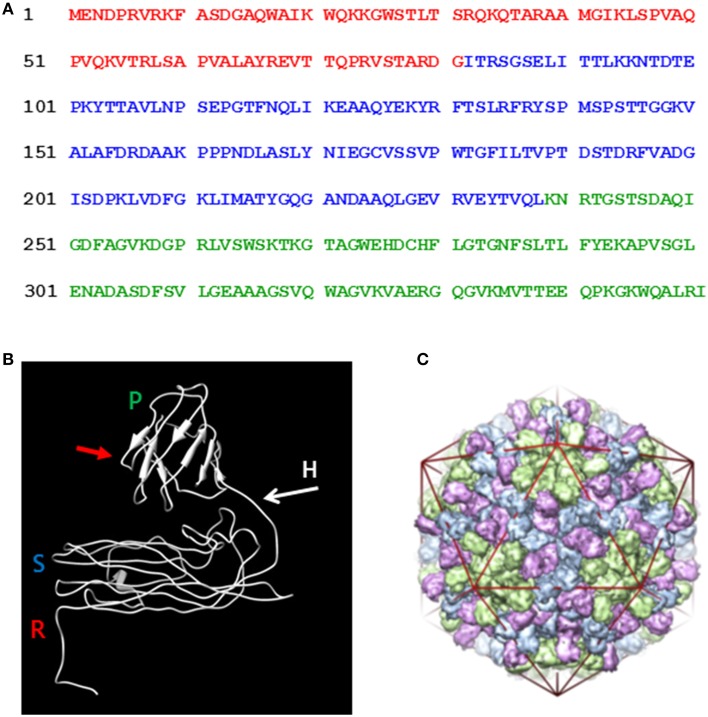
**Amino acid sequence and the folded structure of TCV coat protein. (A)** The coat protein amino acid sequence (after Bakker et al., 2012) indicating the random (red), shell (blue), and projecting (green) structural domain sequences. **(B)** The position of R, random; S, shell; and P, projecting structural domains are indicated in the mature folded coat protein along with the flexible hinge region (H) located between the S and P domains. Red arrow indicates the location at which the hepatitis B epitope MIDIDPYKEFG was inserted into the P domain. **(C)** The TCV *T* = 3 capsid structure (reproduced from Bakker et al., 2012). The three conformational forms of the paired coat protein sub units, green at the five-fold axes and blue with pink at the three-fold axis. The icosahedron in red highlights the locations of the symmetry axis in the capsid structure.

In view of the success with the production of CPMV VLPs in plants, we wished to see if a similar approach could be used to produce VLPs from another plant virus, *turnip crinkle virus* (TCV). TCV is a member of the *carmovirus* genus of the *Tombusviridae* family and encapsidates its RNA genome of 4054 nucleotides into a spherical structure that comprises of 180 units of a single type of coat protein arranged with *T* = 3 symmetry. The advantages of TCV over CPMV are that swollen forms of the particle can be obtained and an *in vitro* assembly system is available (Sorger et al., 1986) potentially allowing the incorporation of larger molecules than is possible with CPMV. Indeed, anionic polyacids have be successfully loaded into protein cages derived from particles of *Hibiscus chlorotic ringspot virus*, a related *carmovirus* (Ren et al., 2006). TCV was one of the first viruses to have its structure determined by crystallography (Hogle et al., 1986) and its swollen capsid structure was recently reported (Bakker et al., 2012). Each TCV coat protein subunit of 38 kDa (351 amino acids, Figure 1A) is folded into three distinct domains. At the N-terminus is the structure-less random (R) domain which interacts with the viral genome on the inner surface of the particle. Next is the shell (S) domain, which forms the continuous protein layer around the viral genome followed, at the C-terminus, by the projecting (P) domain, (Figure 1B). To initiate assembly, pairs of coat protein molecules associate with one another via their S and P domains to form dimers, which subsequently form the body of the capsid shell; depending upon their location in the capsid shell, the dimers are able to adopt one of three distinct structural conformations (Figure 1C). Concurrently, specific sequences of the viral genome interact with the coat protein R domain thereby driving the encapsidation of the viral genome. The flexibility that permits the dimers to adopt three different conformations is provided by the hinge region, the amino acid sequence between the S and P domains. In the current study we have transiently expressed TCV coat protein and its derivatives using the pEAQ transient expression system (Sainsbury et al., 2009) in order to develop an understanding, in the absence of viral infection, as to what modifications can be tolerated by the TCV coat protein while still allowing the formation of TCV-based capsid structures.

## Materials and methods

### Viruses

The DNA sequence of TCV-M (Oh et al., 1995) cloned in pBin61 (35S-TCV; Thomas et al., 2003), was used to infect *Nicotiana benthamiana*. The TCV-M coat protein nucleotide sequence (Bakker et al., 2012) with the GenBank accession code HQ589261 was used throughout this study. CPMV and CPMV eVLPs were made as described by Saunders et al. (2009).

### Molecular cloning

**Figure 2:**
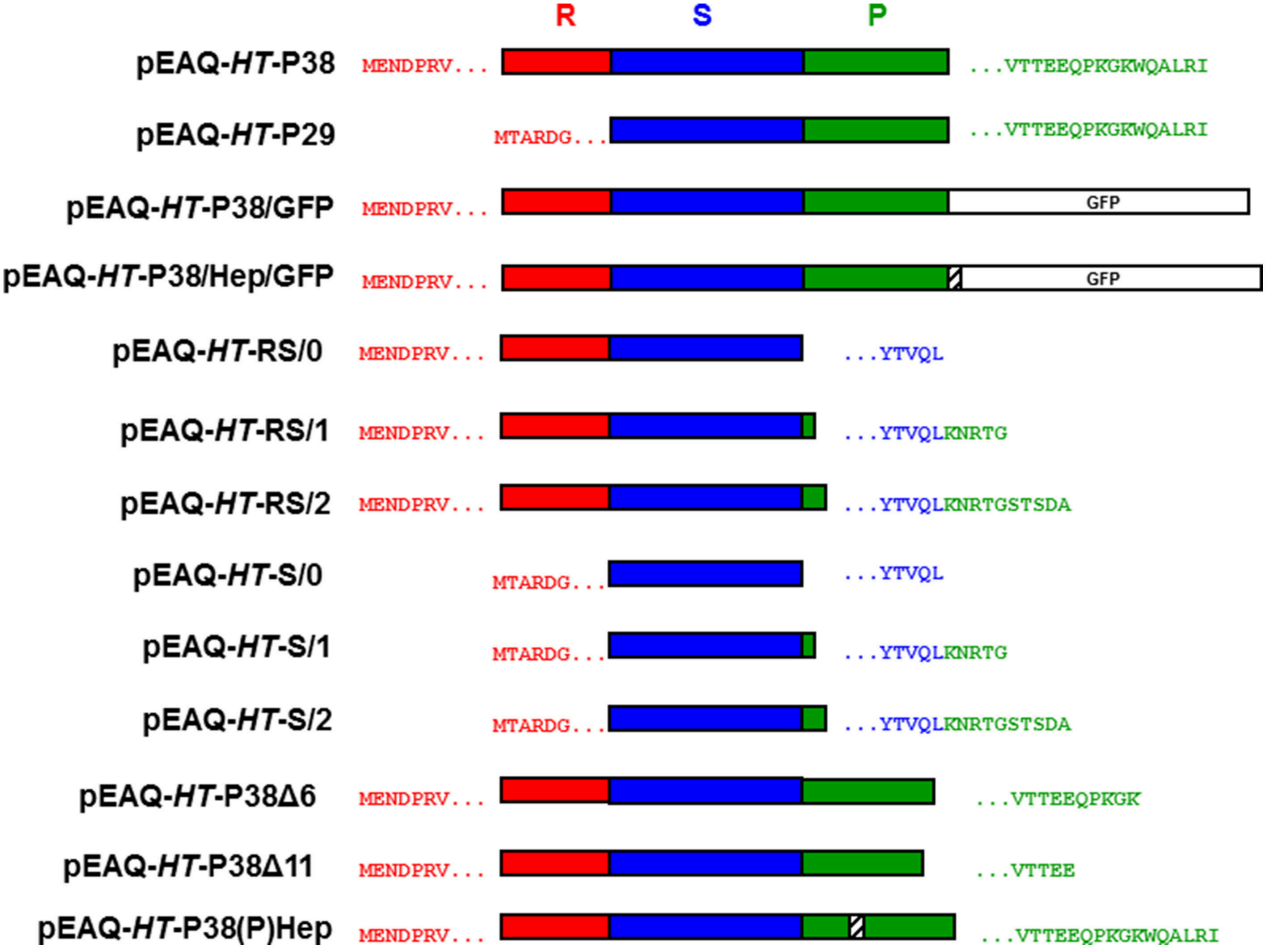
**Schematic representation of the infiltrated coat protein gene constructions**. R, random; S, shell; and P, projecting structural domains within the folded TCV coat protein sequence. The amino acid sequences after the initiating methionine are shown in red with the C-terminal TCV sequence in green and/or blue. Open white box, GFP. Striped box, the location of the inserted hepatitis B epitope MIDIDPYKEFG amino acid sequence.

The nucleotide sequences of the oligonucleotide primers, used in the PCR, to obtain the various gene constructions are listed in Table 1. The coat protein cistron (P38) DNA was derived from 35S-TCV by PCR amplification using primers KS 35 and KS 36. The resulting DNA was cloned utilizing the BC clonase reaction into the Gateway entry plasmid, pDONR207 to create pDON-TCVCP. Subsequently, the Gateway LR reaction was used to transfer the insert of pDON-TCVCP (and all other entry clones) to pEAQ-*HT*-DEST1 (Sainsbury et al., 2009) to yield pEAQ-*HT*-P38 (Figure 2).

**Table 1:** **The sequence of the cloning oligonucleotides**.

**Primer**	**Sequence**
KS 35	5′GGGACAAGTTTGTACAAAAAAGCAGGCTTAATGGAAAATGATCCTAGAGTC
KS 36	5′GGGGACCACTTTGTACAAGAAAGCTGGGTTTACTAAATTCTGAGTGCTTGC
KS 53	5′GGGGACAAGTTTGTACAAAAAAGCAGGCTTAATGACTGCCAGGGACGGCATAAC
KS 70	5′GGGGACCACTTTGTACAAGAAAGCTGGGTTTTCTATTTACCCTTTGGCTGCTCCTC
KS 69	5′GGGGACCACTTTGTACAAGAAAGCTGGGTTTTCTACTCCTCAGTTGTGACCATTTTC
KS 66	5′GGGGACCACTTTGTACAAGAAAGCTGGGTTTTCTAGAGCTGCACGGTGTACTC
KS 67	5′GGGGACCACTTTGTACAAGAAAGCTGGGTTGTCTAGCCAGTTCTGTTCTTGAG
KS 68	5′GGGGACCACTTTGTACAAGAAAGCTGGGTTTGCTAGGCGTCGCTGGTTGAGC

Primers KS 53 and KS 36 were used to clone the amino-terminal deleted P30 form of the TCV coat protein by the same means to finally yield pEAQ-*HT*-P29. The inserted initiating methionine codon was immediately upstream of the amino acid sequence TARDGITR located within the TCV coat protein (Figure 2).

A DNA sequence comprising of the C-terminal portion of the TCV coat protein including a unique *Hind*III site, the hepatitis B core antigen (HbcAg) antigenic site- MDIDPYKEFG and flanking restriction sites was synthesized (GeneArt) and subsequently cloned into pDON-TCVCP to yield pDON-TCVCP-HEP. DNA derived by PCR amplification of the GFP gene in pEAQ-*HT*-GFP (Sainsbury et al., 2009) was cloned using the appropriate restriction sites into pDON-TCVCP-HEP to yield pDON-TCVCP-GFP and pDON-TCVCP/HEP/GFP. Finally the Gateway LR reaction was used to transfer the insert of these plasmids to pEAQ-*HT*-DEST1 (Sainsbury et al., 2009) to yield pEAQ-*HT*-P38/GFP and pEAQ-*HT*-P38/Hep/GFP, respectively (Figure 2).

Primers KS 70 and KS 69 were used, as described above, with primer KS 35 to generate TCV coat protein constructs that lacked either 6 C-terminal amino acids terminating at MVTTEEQPKGK^*^ (pEAQ-*HT*-P38Δ6) or 11 C-terminal amino acids terminating at MVTTEE^*^ (pEAQ-*HT*-P38Δ11) within the TCV coat protein sequence (Figure 2).

A DNA sequence that incorporated the C-terminal portion of the TCV coat protein, including a unique *Hind*III site, and the amino acid sequence MDIDPYKEFG inserted between amino acids 272 and 273 of the TCV coat protein sequence, (Figure 1A) and flanking restriction sites, was synthesized (GeneArt) and subsequently cloned into pDON-TCVCP to yield pDON-TCVCP-(P)Hep. The Gateway LR reaction was used to transfer the insert of this plasmid into pEAQ-*HT*-DEST1 (Sainsbury et al., 2009) to yield pEAQ-*HT*-P38(P)Hep (Figure 2).

Primers KS 66, KS 67, and KS 68, were used as described above with primer KS 35 to generate TCV coat protein gene constructs that consisted of just its R and S domains, pEAQ-*HT*-RS/0, pEAQ-*HT*-RS/1, and pEAQ-*HT*-RS/2. Primers, KS 66, KS 67, and KS 68 were used with primer KS 53 to generate TCV coat protein gene constructs that consisted of just the S domain, pEAQ-*HT*-S/0, pEAQ-*HT*-S/1, and pEAQ-*HT*-S/2. For these gene constructions (with KS 66, KS 67, and KS 68), the coat protein sequence terminated at - YTVQL^*^, YTVQLKNRTG^*^, or YTVQLKNRTGSTSDA^*^, respectively, (Figure 2).

The sequence of all the resulting gene constructs, both in pDONR207 and in pEAQ-*HT*-DEST1 were subsequently confirmed by DNA sequencing analysis (Eurofins Genomics). Verified pEAQ-*HT* clones were used to transform *Agrobacterium tumefaciens* LBA4404 and were subsequently used for plant infiltration experiments (Sainsbury et al., 2014).

### Analysis of TCV-inoculated and plant-infiltrated material

*Agrobacterium tumefaciens* infiltrated leaf tissue and TCV inoculated plant leaves were harvested 6 or 7 days post infiltration and homogenized in three volumes of extraction buffer (1 mM MgSO_4_, 1 mM NaPO_4_ pH 7.4). After squeezing through Miracloth (Calbiochem), the extracts were clarified by centrifugation at 12,000 × g for 30 min at 4°C. Thirty-three grams of 30% (w/v) PEG 6000 and 0.8 mL of 5M NaCl per 100 mL of resulting supernatant were combined and stirred overnight in a cold room. Protein was sedimented at 14,460 × g for 30 min at 4°C and the pellet was re-suspended in extraction buffer and further clarified at 27,000 × g for 20 min at 4°C. TCV and virus-like particles in the resulting supernatant were recovered by centrifugation at 118,700 × g for 3 h at 4°C, re-suspended in extraction buffer and loaded onto 10–50% (w/v) sucrose step gradients in extraction buffer and centrifuged at 136,873 × g for 1.5 h at 4°C. Material derived from pEAQ-*HT*-P38/GFP and pEAQ-*HT*-P38/Hep/GFP infiltrated plant leaves were not subjected to precipitation with PEG. After the initial clarification step at 12,000 × g for 30 min at 4°C, the protein extract was subjected to 30–60% (w/v) sucrose step gradient centrifugation at 166,880 × g for 2 h at 4°C. After fractionation, selected fractions were dialyzed overnight in H_2_O prior to TEM analysis. TCV and virus-like particles recovered after centrifugation at 118,700 × g were subjected to centrifugation for 24 h at 209,627 × g (10°C) in CsCl gradient formed of equal volumes of 42, 49, 57, and 65% (w/v) CsCl in 10 mM NaPO_4_ pH7.0. The resulting separations were photographed with white light directly above. Green fluorescent protein was detected and photographed in leaves under UV light.

### Phenol extraction and agarose gel analysis

Selected TCV and virus-like particle sucrose gradient fractions that were subsequently dialyzed with H_2_O were subjected to phenol/CHCl_3_ extraction as described by Saunders et al. (2009). RNA recovered by ethanol precipitation was denatured in formamide/formaldehyde and resolved in 1.35% (w/v) agarose gels containing formaldehyde and ethidium bromide in 100 mM MOPS, 40 mM acetic acid buffer pH 7.0. Untreated virus and virus-like particles were separated in 1.2% agarose gels in Tris-borate buffer pH 7.4. Particles were visualized by staining with ethidium bromide or Coomasie Brilliant Blue, the latter de-stained with 20% (v/v) methanol, 7.5% (v/v) acetic acid.

### SDS-PAGE and western blot analysis

Protein extracts and gradient fractions were analyzed by electrophoresis in 12% NuPAGE Bis-Tris gels resolved with MOPS buffer (Life Technologies). Instant Blue stain (Expedeon Ltd) was used to visualize the protein bands. Western blot analyses of Hep fusion proteins were performed using a monoclonal primary antibody against the HBcAg protein epitope (10E11, [Abcam]) followed by detection with a goat anti-mouse secondary antibody conjugated to horseradish peroxidase, and developed using the chemiluminescent substrate ECL plus (Amersham Pharmacia). A GFP antibody-HRP conjugate (A10260 Invitrogen) was used to detect GFP in western blots.

### Negative-stained transmission electron microscopy

Particle preparations were spotted onto carbon-coated copper grids and negatively stained with 2% (w/v) uranyl acetate. The grids were examined using a Tecnai 20 transmission electron microscope.

## Results and discussion

### Expression of full-length and modified forms of the TCV coat protein

**Figure 3:**
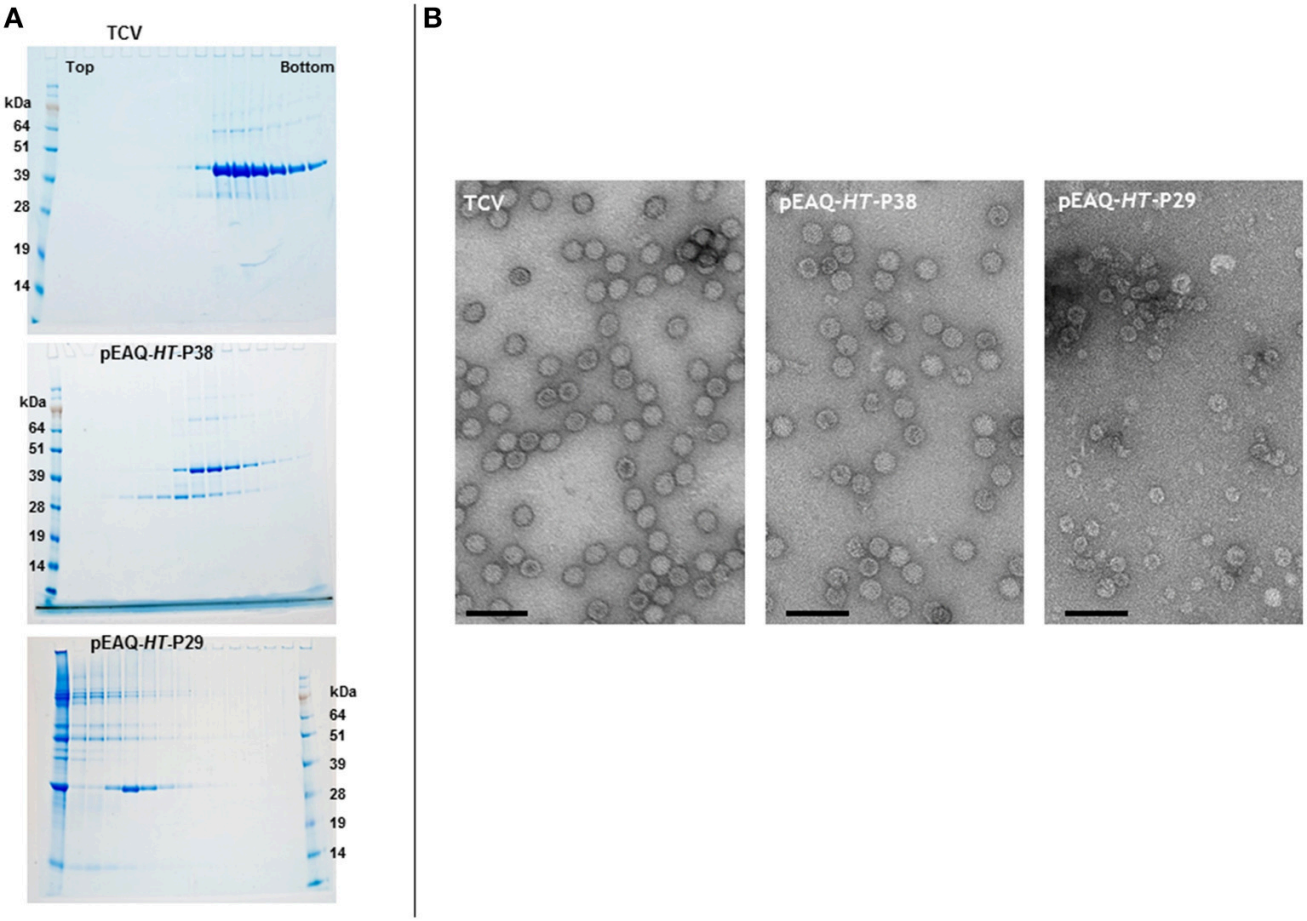
**Sucrose gradient and transmission electron microscopy analysis of the expression of gene constructions in infiltrated leaves**. **(A)** 10–50% (w/v) sucrose gradients show the presence of intact TCV coat protein with a gel migration of 38 kDa, upper panel. The smaller product with a gel migration of 30 kDa results from either the expression of the TCV coat protein or the expression of just the S and P domains of the coat protein. Upper panel, TCV; middle, pEAQ-*HT*-P38; and lower panel, pEAQ-*HT*-P29 expression. Protein ladder, SeeBlue® Plus 2, pre-stained protein standard catalog number: LC5925 (Invitrogen) **(B)** Transmission electron microscopy of the peak fractions of the samples shown in **(A)**. Scale bar = 100 nm.

Denaturing polyacrylamide gel analysis and Instant Blue staining of extracts from *Nicotiana benthamiana* leaves infiltrated with pEAQ-*HT*-P38 containing the sequence of the full-length TCV coat protein (Figure 2), revealed the presence of a protein that had gel migration equivalent to that of TCV coat protein (P38) which had been extracted from purified TCV particles (data not shown). The identity of this protein as the TCV coat protein was confirmed by subsequent MALDI-TOF analysis. To test whether the transiently expressed P38 can form TCV-like particles, extracts from infiltrated leaves were subjected to the TCV extraction and purification protocol and the resulting material analyzed on 10–50% (w/v) sucrose gradients. As a control, authentic TCV particles produced via infection and extracted in the same way were analyzed in parallel. Fractions from the gradients were analyzed by electrophoresis on denaturing polyacrylamide gels followed by Instant Blue staining (Figure 3A, upper two panels). In the case of authentic TCV, all the P38 sedimented toward the bottom of the gradient consistent with the subunits being incorporated into assembled virus particles. In addition, a small amount of a 30 kDa (P30) and an 80 kDa protein (P80) co-sedimented with P38. P30 has previously been identified as a proteolysed version of P38 consisting of just the S and P domains while P80 represents a covalent dimer of P38 (Golden and Harrison, 1982; Sorger et al., 1986; Stockley et al., 1986; Bakker et al., 2012). The same three proteins were found when particles were isolated from leaf tissue infiltrated with pEAQ-*HT*-P38 but their distribution was somewhat different in the gradient: P38 and P80 occurred in more slowly sedimenting fractions than was found when virus was analyzed and there was an increased amount of P30 whose sedimentation overlaps with that of the other two proteins but which also occurs in more slowly sedimenting fractions. MALDI-TOF analysis of P30 derived from pEAQ-*HT*-P38 confirmed that it contains only peptides that are found in the S and P domains of P38, (data not shown), consistent with it lacking the R domain. This slower sedimentation is consistent with the previous suggestion that TCV coat protein lacking its R domain (P30), can form *T* = 1 capsid structures (Sorger et al., 1986). These observations suggest the protein expressed from pEAQ-*HT*-P38 is capable of forming VLPs with lower *S* value than native TCV and that the protein undergoes the same pattern of dimerization and proteolysis as the coat protein in the native virus. Furthermore, the results confirm that, in contrast to CPMV eVLPs, no other, additional, virus-derived proteins are required for the formation of TCV VLPs.

To confirm that the expression of TCV coat protein, in the absence of a virus infection, results in the formation of VLPs, the fractions from the authentic TCV and pEAQ-*HT*-P38 gradients containing P38 were dialyzed and examined by transmission electron microscopy (TEM). In both cases, particles with a diameter of approximately 28–32 nm, characteristic of TCV particles could be seen (Figure 3B, left two panels). This clearly indicates that P38 transiently expressed in plants is capable of efficiently forming *T* = 3 VLPs. The fact that these VLPs sediment more slowly than authentic TCV particles, despite having a similar morphology, is consistent with them having a lower RNA content. The ability of P30, produced by proteolysis of P38 to form more slowly sedimenting (presumably *T* = 1) VLPs, suggests that it should be possible to deliberately alter the size of TCV VLPs through the genetic deletion of the R domain. To test this, pEAQ-*HT*-P29, a gene construct that lacked the amino-terminal R domain of the TCV coat protein, was constructed and infiltrated into leaves. The initiation codon selected for this construct was based on the identification of the N-terminus of P30 present in TCV preparations (Bakker et al., 2012). Figure 3A, lower panel shows that under identical sedimentation conditions, P30 alone forms particles that sediment more slowly than those formed by P38 products. Observation of these fractions under the TEM (Figure 3B, right-hand panel), revealed the presence of particles that are smaller, with an approximate diameter of between 22 and 25 nm, than the particles present in plant leaves infiltrated with pEAQ-*HT*-P38 or authentic TCV particles. This size difference indicates that P30 is able to exclusively direct the formation of smaller *T* = 1 particles whereas P38 orchestrates the formation of the larger *T* = 3 particles. This indicates that it is possible to deliberately alter the size and symmetry of TCV-based VLPs by alterations to the coat protein. The total yield of particles after sucrose gradient purification was estimated to be approximately 5.0 and 1.5 mg/Kg wet weight infiltrated leaf material for *T* = 3 and *T* = 1 particles, respectively.

### Plant-expressed TCV coat protein encapsidates heterogeneous RNA

**Figure 4:**
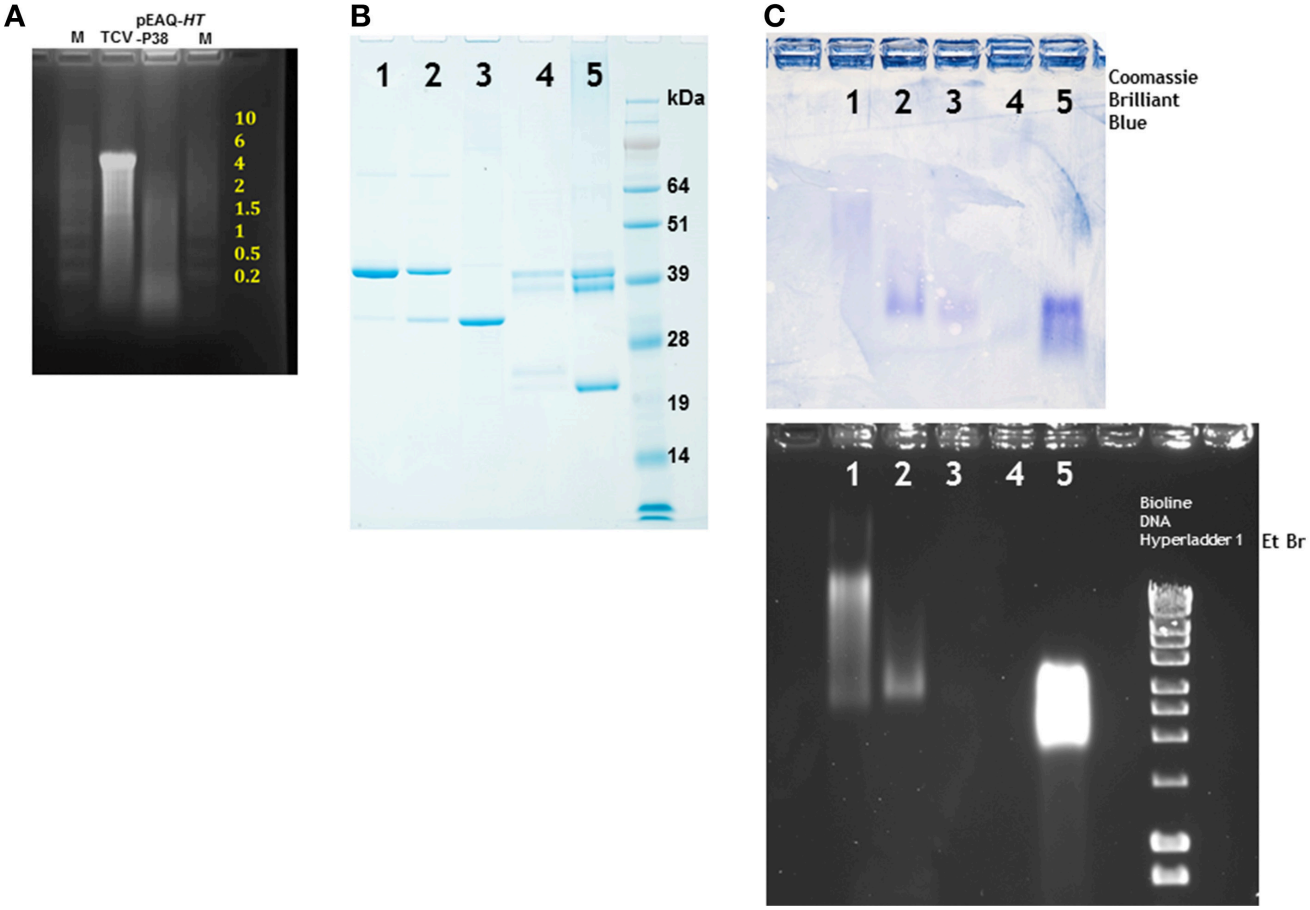
**Denaturing electrophoresis of RNA extracted from particles and particle migration under non-denaturing conditions**. **(A)** Denaturing agarose electrophoresis of nucleic acid extracted from TCV and particles formed by the infiltration of leaves with pEAQ-*HT*-P38. Yellow numbering denotes the size of the RNA (kb) ladder in lane M, catalog number 15623-200 (Invitrogen). **(B)** Migration of denatured viral proteins in 12% NuPAGE gel. **(C)** Separation of particles in a non-denaturing 1.2% agarose gel. Upper panel stained with Coomassie Brilliant Blue, the lower panel stained with ethidium bromide. 1, TCV; 2, pEAQ-*HT*-P38; 3, pEAQ-*HT*-P29; 4, CPMV eVLPs; 5, CPMV. Right lane lower panel, a commercially available DNA ladder (Bioline) confirming that the gel was stained with ethidium bromide.

The RNA contents of sucrose gradient purified authentic TCV or VLPs produced by infiltration with pEAQ-*HT*-P38 were analyzed by denaturing agarose gel electrophoresis. While the RNA extracted from TCV gave a predominant band at about 4 kb, a size consistent with it representing the viral genomic RNA, the RNA isolated from the particles produced by infiltration with pEAQ-*HT*-P38 is heterogeneous with a predominant size range between approximately 100 nucleotides and 1500 nucleotides (Figure 4A). This finding indicates that when full-length genomic RNA is present in a cell, as is the case with a TCV infection, it is preferentially packaged but that in its absence other cellular RNAs can be encapsidated. Clearly the R domain has the ability to interact with and induce the packaging of single-stranded RNA that is not of virus origin. Several putative RNA packaging signals have been identified on TCV genomic RNA, some of which lie within the polymerase gene and some in the coat protein sequence (Wei et al., 1990; Wei and Morris, 1991; Qu and Morris, 1997). The fact that RNA of the size corresponding to the mRNA directing the synthesis of coat protein (approximately 1700 nucleotides) could not be identified in the RNA extracted from P38 particles, despite this RNA containing some of the previously identified packaging signals, is consistent with the suggestion that RNA size is also important for efficient packaging (Qu and Morris, 1997).

Sucrose gradient purified particles were further analyzed by subjecting the *T* = 3 and *T* = 1 particles derived by infiltration of pEAQ-*HT*-P38 and pEAQ-*HT*-P29, respectively, to electrophoresis in non-denaturing agarose gel followed by specific staining, independently, for either protein or nucleic acid. The relative migration of different particles is determined by both their size and overall charge which is, in turn, related to their RNA content (Steinmetz et al., 2006). As controls TCV, CPMV and RNA-free empty CPMV virus-like particles (eVLPs; Saunders et al., 2009) were analyzed in parallel. To ensure the samples contained detectable levels of protein, they were first analyzed by electrophoresis on denaturing polyacrylamide gels. As expected, the TCV sample contained mainly P38 with small amounts of P30 and P80 (Figure 4B, lane 1) while the VLPs produced by infiltration with pEAQ-*HT*-P38 contained the same proteins but with an increased proportion of P30 (Figure 4B, lane 2). By contrast the VLPs produced by infiltration with pEAQ-*HT*-P29 contained predominantly P30 (Figure 4B, lane 3). The intensity of the band representing the major component (P38 or P30) was similar in each case. CPMV and CPMV eVLPs gave a characteristic pattern of bands representing various forms of the Large (L) and Small (S) coat proteins on the denaturing gel (Sainsbury et al., 2014), although the intensity of staining was considerably less in the case of eVLPs (Figure 4B, lanes 4 and 5) indicating there was less protein the sample. Staining of a non-denaturing agarose with Coomassie Brilliant Blue will reveal the position of all types of particle after electrophoresis by specifically staining the capsid protein (Figure 4C, upper panel), while ethidium bromide staining should detect only those particles containing encapsidated nucleic acid. As anticipated, both the control CPMV particles and CPMV eVLPs stained with Coomassie Brilliant Blue (Figure 4C, lanes 4 and 5), although the staining of the eVLP sample was less intense, consistent with lower amount of protein seen in the denatured sample. By contrast, only the virus particles containing RNA stained with ethidium bromide (Figure 4C, lane 5). The staining of the virus particles was so intense that if the eVLPs had contained nucleic acid this would have been readily visualized despite the lower amount of material loaded. TCV and P38 particles stained with both Coomassie Brilliant Blue and ethidium bromide but the relative intensity of ethidium bromide staining was less with the P38 particles, a result consistent with their having a lower RNA content than authentic TCV particles (Figure 4C, lanes 1 and 2). Furthermore the particles migrated to very different positions in the gel, with the P38 particles migrating more quickly than those of authentic TCV, despite their having similar morphology as indicated by TEM (Figure 3B). Particles derived from the infiltration of leaves with pEAQ-*HT*-P29 could be detected only with Coomassie Brilliant Blue, indicating that these have not encapsidated significant amounts of RNA, a property shared by the CPMV eVLPs (Figure 4C, lanes 3 and 4). The lack of any detectable RNA within the particles formed following the infiltration of pEAQ-*HT*-P29 is consistent with the fact that P30 does not contain those amino acids (the R domain) that are known to bind to RNA (Sorger et al., 1986). The ability to control the size and RNA content of TCV VLPs by manipulating the coat protein sequence could be a particularly useful property of the particles in terms of bionanotechnology.

### Expression of TCV coat protein fused to GFP

**Figure 5:**
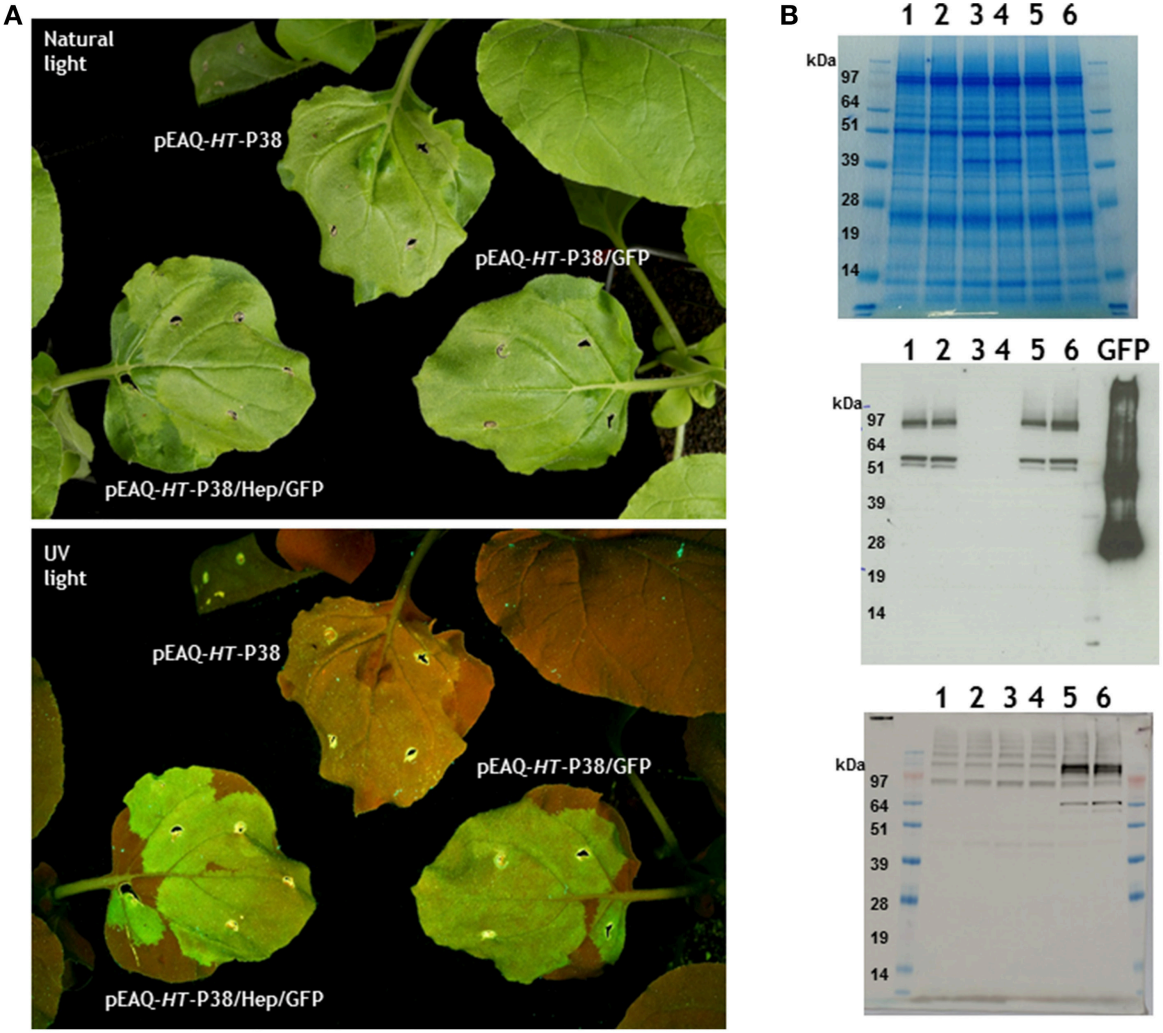
**Expression of TCV coat protein-GFP gene fusions in infiltrated plant leaves. (A)** Visualization of infiltrated leaves under natural or UV light. **(B)** Gel analysis of proteins from infiltrated leaves. 1 and 2, pEAQ-*HT*-P38/GFP; 3 and 4, pEAQ-*HT*-P38; 5 and 6, pEAQ-*HT*-P38/Hep/GFP. Upper panel stained with Instant Blue. Western blot analysis; middle panel antibody against GFP, GFP control is 1μg of a hepatitis B coat protein/GFP hybrid protein (Peyret et al., 2015). Lower panel monoclonal primary antibody against the hepatitis HBcAg protein epitope.

One potential use of TCV VLPs is the presentation of foreign sequences on the particle surface. Previous work on the structurally related *Tombusvirus, Tomato bushy stunt virus* (TBSV), showed that peptides could be fused to the C-terminus of the coat protein so that they are expressed on the P domain without abolishing virus or VLP assembly (Joelson et al., 1997; Kumar et al., 2009). The neutralizing HIV-1 2F5 epitope was recently expressed on *Artichoke mottled crinkle virus* surface, a virus also of the *Tombusvirus* genus (Arcangeli et al., 2014). We therefore decided to investigate whether it would be possible to present whole proteins, as opposed to peptides, on the surface of plant-expressed TCV VLPs. As a starting point the reporter gene GFP was cloned in-frame at the C-terminus of the coat protein to generate pEAQ-*HT*-P38/GFP. GFP forms a tight compact barrel-like structure that is resistant to proteolytic attack and provides a useful way of tracking expression levels. An additional gene construct was also made in which the hepatitis B core epitope, MDIDPYKEFG, derived from the N-terminus of the hepatitis B core antigen (HBcAg) was inserted in frame between the TCV CP and the reporter gene to yield pEAQ-*HT*-P38/Hep/GFP. This was done because this region of HBcAg is unstructured, and could therefore serve as a linker, and a commercial monoclonal antibody to this epitope is readily available. The infiltration of such gene constructs was intended to lead to the synthesis of TCV VLPs that are coated with GFP. As a control, leaves were infiltrated with pEAQ-*HT*-P38 which lacks the GFP sequence. Five days after infiltration, green fluorescence was evident under UV illumination in those leaves that had been infiltrated with pEAQ-*HT*-P38/GFP or pEAQ-*HT*-P38/Hep/GFP, but not those infiltrated with pEAQ-*HT*-P38 (Figure 5A). The presence of fluorescence indicates that translation of the entire fusion protein has occurred.

At 7 days post-infiltration total soluble protein from infiltrated leaves was analyzed by electrophoresis on denaturing polyacrylamide gels followed by Instant Blue staining. A product corresponding to the migration of TCV P38 could be seen in extracts from those leaves that had been infiltrated with pEAQ-*HT*-P38 (Figure 5B, Top panel lanes 3 and 4) but not in extracts from leaves infiltrated with the GFP fusion gene constructions (Figure 5B, Top panel, lanes 1, 2, 5, and 6). The GFP fusion proteins could not be seen after Instant blue staining suggesting they accumulated to a lower level or were obscured by other plant proteins running at similar position. Western blotting with anti-GFP antibody detected protein products at around 62 kDa in extracts of leaves infiltrated with pEAQ-*HT*-P38/GFP or pEAQ-*HT*-P38/Hep/GFP, whose migration is consistent with the generation of a TCV coat protein-GFP fusion protein (Figure 5B, middle panel, lanes 1, 2, 5, and 6). No signal was seen in extracts infiltrated with pEAQ-*HT*-P38 which lacks the sequence of GFP. The smaller product, migrating just below the 62 kDa band (by approximately some 9 kDa) is likely to represent the coat protein-GFP fusion protein from which the amino-terminal R domain has been cleaved. The expression of both pEAQ-*HT*-P38/GFP and pEAQ-*HT*-P38/Hep/GFP also resulted in the generation of products of size greater than approximately 100 kDa which reacted with anti-GFP serum. These may have arisen as the result of the dimerization of two coat protein units generated during the formation of virus particles i.e., representing the P80 covalent dimer carrying GFP. The lower panel of Figure 5B shows the detection of the TCV coat protein/hepatitis epitope/GFP gene fusion product with the monoclonal antibody directed to the hepatitis B core epitope amino acid sequence in extracts from leaves infiltrated with pEAQ-*HT*-P38/Hep/GFP but not with pEAQ-*HT*-P38/GFP or pEAQ-*HT*-P38. The faint higher molecular weight bands seen with the hepatitis B core antibody occur in all tracks and are therefore due to a non-specific reaction between this antibody and unknown plant proteins. Again, this confirms the synthesis of the fusion gene construction product, the pattern of products being similar to that found when anti-GFP antibodies were used.

**Figure 6:**
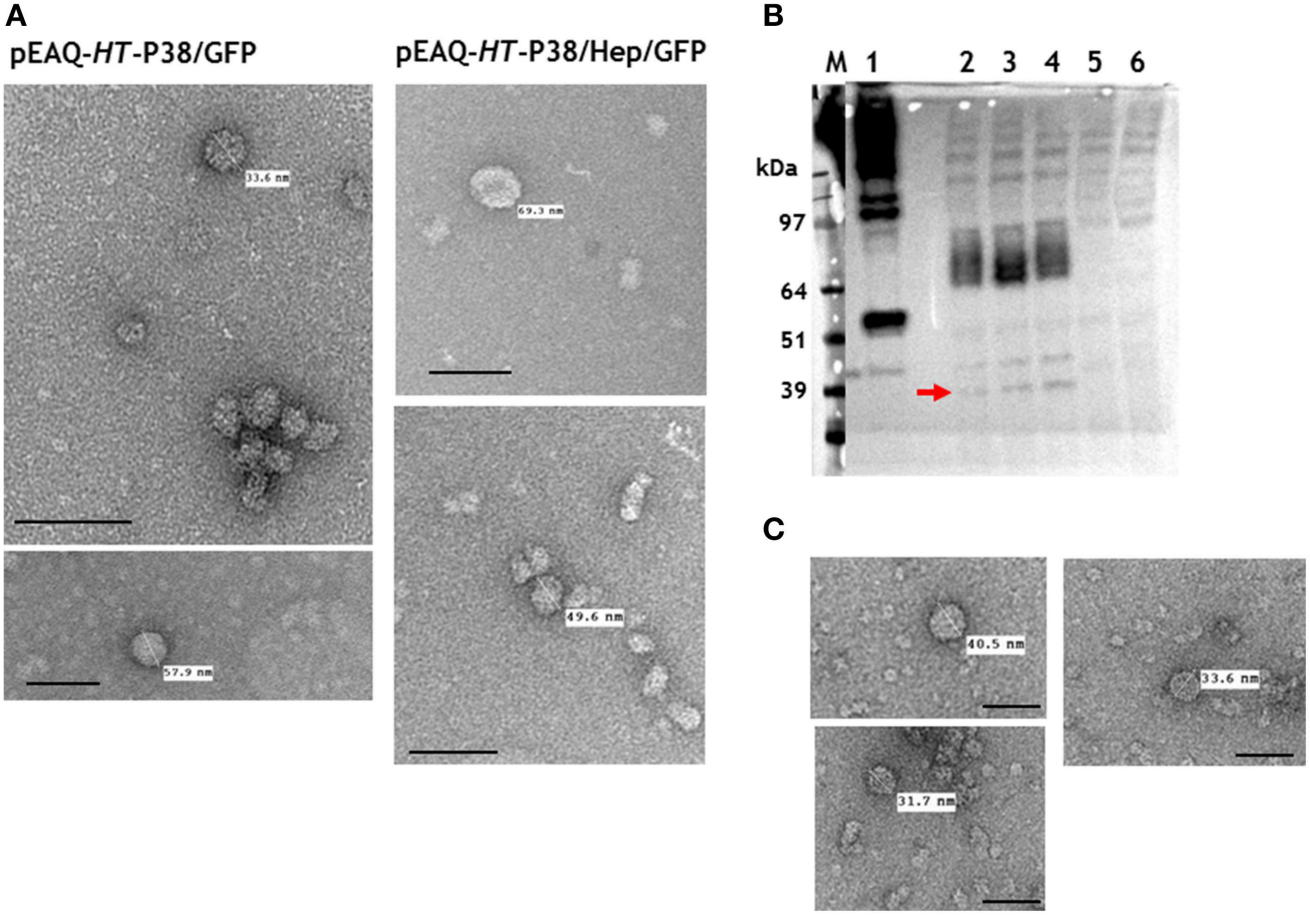
**Particle and gel analysis of samples from leaves infiltrated with gene constructs engineered with modifications to TCV coat protein**. **(A)** Transmission electron microscopy (TEM) of particles formed following the expression TCV coat protein/GFP gene fusions. **(B)** Western blot detection (with monoclonal primary antibody against the hepatitis HBcAg protein epitope) of TCV coat protein possessing the hepatitis HBcAg epitope. 1, pEAQ-τGFP (Peyret et al., 2015); 2, 3, and 4, pEAQ-*HT*-P38(P)HEP gene constructs; 5, TCV; 6, mock infiltrated leaves. The red arrow indicates the detection of hybrid coat protein. **(C)** TEM of gradient purified particles formed following the expression of pEAQ-*HT*-P38(P)HEP. Scale bar = 100 nm.

Any potential particles generated by infiltration with pEAQ-*HT*-P38/GFP or pEAQ-*HT*-P38/Hep/GFP were isolated from infiltrated leaves using the standard TCV protocol and analyzed by centrifugation on 10–60% (w/v) sucrose gradients. Selected fractions, were examined by TEM. Irregular-shaped particles of over 30 nm diameter were evident in both preparations (Figure 6A) which were more abundant in the samples prepared from leaves infiltrated with the construct (pEAQ-*HT*-P38/GFP) not possessing the hepatitis B core epitope. This suggests that the expressed modified TCV coat protein is able to fold and form particles albeit not with the same high efficiency as wild-type coat protein. From the sucrose fractions, we estimate that the yield of the GFP containing VLPs to be less than 0.1% of that of the *T* = 3 particles derived from infiltration with pEAQ-*HT*-P38. These results indicate that the expression of GFP at the C-terminus of P38 hinders particle formation, possibly by causing steric hindrance between adjacent P domains.

### An intact P domain is essential for capsid formation

A possible way to increase the ability of TCV VLPs to display heterologous proteins would be to replace the P domain with the required sequence. Though the P domain does not form the main structural element of the capsid, it may play an important role in particle assembly by enabling P38 to dimerise (Hogle et al., 1986; Sorger et al., 1986). To investigate the necessity of the P domain for VLP assembly, two series of constructs were made in which the entire P domain was deleted but varying amounts of the hinge region were retained (Figure 2). One series retained both the R and S domains (pEAQ-*HT*-RS/0 to—RS/2) while the other was based on just the S domain (pEAQ-*HT*-S/0 to—S/2). Following infiltration of the constructs, no VLPs could be detected, consistent with the importance of the P domain for particle assembly. A further two constructs that lacked either the C-terminal 6 (pEAQ-*HT*-P38Δ6) or 11 amino acids (pEAQ-*HT*-P38Δ11) of P38 (Figure 2), were made and infiltrated in *N. benthamiana* leaves. Again, no VLPs were generated suggesting that amino acids at the C-terminus of the P domain play a vital role in virus assembly. The abolition of assembly through deletion of all or part of the P domain effectively precludes the use of such deletions to increase the space available for the presentation of heterologous sequences.

To determine whether an alternative site in the P domain can be used to present foreign sequences, a gene construct, pEAQ-*HT*-P38(P)Hep, containing the hepatitis B core epitope MIDIDPYKEFG, inserted between amino acids 272 and 273 of P38, was created (Figure 2). This was designed so that the foreign sequence would be presented in a loop on the opposite face of the P domain to that which is involved in dimer formation. The presence of this hybrid protein in extracts of leaves infiltrated with pEAQ-*HT*-P38(P)Hep was examined by western blot analysis of total protein extracts using the antibody directed against this epitope (Figure 6B). The most abundant product was represented by a broad band migrating with a size between 97 and 64 kDa (Figure 6B, lanes 2, 3, and 4), which could represent the fused dimeric form of the modified coat protein. A relative smaller amount of a protein of the size expected for a monomeric form of the modified form of P38 could also be seen (Figure 6B, arrowed). VLP preparations were carried out as described above and the resultant material analyzed by centrifugation on a 10–50% (w/v) sucrose gradient. Gradient fractions that corresponded to P38 VLPs, run on a duplicate gradient, contained very few viral particles when examined by TEM (Figure 6C). The difference in detection between monomeric and the fused dimeric forms of the hepatitis B core epitope-containing coat protein (Figure 6B), equivalent to P80, suggests that this modified dimeric form is very stable but is not readily incorporated into capsid structures and therefore accumulates. The anti-hepatitis B monoclonal antibody to the epitope sequence was unable to detect the hybrid particles in the ultracentrifuged gradient fractions (data not shown) again indicating that formation of these hybrid viral particles was inefficient compared to unmodified P38 VLPs.

Taken together these expression studies confirm that the interaction between two folded coat protein molecules via their P domains is a key step in particle assembly. Thus, modifications to the P domain should involve just the insertion of foreign sequences. Even in this case the yield of assembled particles is considerably reduced, probably due to steric hindrance especially with large inserts such as GFP. A possible solution to this may lie in the creation of mosaic particles containing both the modified and unmodified subunits in an approach analogous to that recently used for HBcAg particles (Peyret et al., 2015).

## Author contributions

Conceived and designed the experiments: KS, GL. Performed the experiments: KS. Analyzed the data: KS, GL. Wrote the paper: KS, GL.

## Funding

This work was supported by the UK Biotechnological and Biological Sciences Research Council (BBSRC) Institute Strategic Programme Grant “Understanding and Exploiting Plant and Microbial Secondary Metabolism” (BB/J004596/1) and the John Innes Foundation.

### Conflict of interest statement

The authors declare that the research was conducted in the absence of any commercial or financial relationships that could be construed as a potential conflict of interest. GL declares that he is a named inventor on patent WO 29087391 A1 which describes the transient expression system used in this manuscript.
